# Supplementary material for: The Importance of Chitosan Coatings in Dentistry
Source: Mar Drugs. 2023 Nov 26;21(12):613. doi: 10.3390/md21120613 (PMC10744558; doi:10.3390/md21120613)
Supplement: Supplementary file 1 [file marinedrugs-21-00613-s001.zip › marinedrugs-2584248-supplementary.pdf]

Table S1. Presentation of the substances and their activity in organism to chitosan-based coatings used in dental surgery.

Substance	Reaction	Author (reference)
Fuoride-doped diopside nanoparticles into chitosan-based coatings	- Highest osteoblast reaction at 60% of cell viability (among 20, 40, 60, 80 of the examined pieces)<br>- Attachment on the surface improves with the amount of particles<br>- Improvement of bioperformance of implants	Karimi et al. [44]
Chitosan coated iron oxide nanoparticles (IONPs) with fluconazole	- Biologically inert<br>- Strenghtens the fluconazole reaction	Paulino-Gonzales et al. [45]; Caldeirao et al [46].
Chitosan coated iron oxide nanoparticles (IONPs) with miconazole (MCZ)	- miconazole activity in IONPs-CS-MCZ superior antibiofilm effect	Arias et al [47]
Chitosan coated iron oxide nanoparticles (IONPs) with fluconazole	- No specific reaction<br>- Reduced minimum inhibitory concentration of fluconazole<br>- Increased antifungal value	de Lima et al. [16]
Chitosan and chitosan+azithromicin	- Strenghtening of azithromicin reaction<br>- suppression of biofilm (Porphyromonas gingivalis) formation	[Anggani et al. [42]
Two degrees of deacetylation (DDA) chitosan	- Promotes bone healing around the implant	[Alnufaiy et al. [43]
Layer by layer (LBL) coating with double layers of chitosan	- Increased biomineralization and osteoblastic potential<br>- Anti-bacterial activity against Streptococcus gordonii	Govindharajulu et al. [15]
Chitosan-ZnO coating	- Effectiveness in fighting Escherichia coli<br>- Improved corrosion resistance<br>- Good cytocompatibility in MG-63 cells	Lin et al. [48]
Bovine serum albumine (BSA) with addition of chitosan	- Delays BSA release form bioactive glass	Liu et al. [49]
Chitosan	- Treatment of gingivitis<br>- Disinfection of dentures and prosthetic devices<br>- quite stable on PMMA	Walczak et al. [50]
Chitosan and hybrid coating ($TiO_2/MoSe_2$/CHI)	- Improved hydrophilicity, biocompatibility, osseointegration	Chai et al. [51]

	- Promoted anti-Streptoccocus mutans reaction	
Chitosan	- Shows a high biocompatibility<br>- Stable to different, extreme physical conditions (low pH) and time	Campos et al. [52]; Kalyoncuoglu et al. [53]
Chitosan	- 15 layers of chitosan coating acts as a low cost GTR membrane	Fernandes et al. [54]
Microsphere based on chitosan-coated alginate (CA) and poly(meth)acrylate-glycerin (PG) with minocycline	- Longer carrier and bacteriostatic effect<br>- No improvement in long-term treatment outcomes	Yoon et al. [55]
Apatite-Wollastonite-Chitosan (AW-Chitosan)	- Positively influences cell proliferation, growth and mineralization<br>- longer drug sustainability did not influence the treatment<br>- longer bacteriostatic effect	Murkherjee et al. [56]
Chitosan coating clotrimazole-composite sandwich nanofibres	- Antifungal (Candida albicans) activity<br>- Longer time of coating results a slower release of clotrimazole<br>- The antifungal effect was generally larger (faster reaction)	Tonglairoum et al. [57]
Chitosan-gold nanoparticles coated titanium implants	- Chitosan increased the volume and density of newly formed bone and the osseointegration of dental implant	Takanche [58]
